# Value of Routine Follow-Up After Pleomorphic Adenoma Surgery

**DOI:** 10.3390/jcm15020656

**Published:** 2026-01-14

**Authors:** Yehonatan Adler, Eyal Yosefof, Ben Giladi, Mor Shukrun, Aviram Mizrachi

**Affiliations:** 1 Department of Otorhinolaryngology—Head and Neck Surgery, Rabin Medical Center, Petach Tikva 4941492, Israel; johnnyadler2@gmail.com (Y.A.);; 2 Gray Faculty of Medicine, Tel Aviv University, Tel Aviv 6997801, Israel; 3 Institute of Oncology, Davidoff Center, Rabin Medical Center, Petach Tikva 4941492, Israel; 4 Department of Otolaryngology—Head and Neck Surgery, Weill Cornell Medical College, Cornell University, New York, NY 14850, USA

**Keywords:** pleomorphic adenoma, benign mixed tumor, follow-up, recurrence, self-discovery

## Abstract

**Objective:** Pleomorphic Adenoma (PA) is the most common benign salivary gland tumor. The need and duration of follow-up post resection is debatable. The aim of our study was to examine the need for routine follow-up after PA resection. **Methods:** Retrospective case-series analysis of adult patients with PA, between 1990 and 2023. Numbers of office examinations, imaging studies, time from first surgery to recurrence and recurrence detection modality were collected. **Results:** Overall, 301 patients had undergone surgery for PA, of which 28 experienced recurrence (9.3%). Mean time from surgery to first recurrence was 119.5 months (range, 33–274). Of the 40 recurrences, 36 were self-discovered (90%), 2 by clinical examination (5%) and 2 by imaging (5%). A total of 618 office examinations and 155 imaging studies were performed. **Conclusions:** Routine examinations and imaging appear to be of low yield in most PA patients, specifically in the first 3 years. Patients should be counseled about self-detection and ominous symptoms necessitating evaluation.

## 1. Introduction

Pleomorphic adenoma (PA) is the most common benign neoplasm of the salivary glands. Largely, the major salivary glands are the site of origin, specifically the parotid gland, but PAs may also arise in minor salivary glands, most commonly in the hard palate.

Most PAs are in a plane superficial to the facial nerve, but approximately 10% originate entirely in the plane deep to the facial nerve (“deep lobe” of the parotid gland).

It is called “pleomorphic” because it possesses both epithelial and mesenchymal components. It is agreed that the tumor originates from an uncommitted reserve cell of the intercalated duct with the potential to differentiate into various types of cells.

On presentation, most patients present with a firm, well demarcated, non-tender round mass in the parotid. Some may report rapid enlargement of the mass, but most of them will report its presence for many years. A full nose–throat examination is extremely important: any deviation from the depiction provided earlier should raise suspicion of a different diagnosis, requiring rapid evaluation. For example: facial nerve weakness; local pain; a mass adherent to the mandible, among others, are ominous signs and symptoms and should raise suspicion for other diagnoses such as malignancy [1].

Standard evaluation consists of fine needle aspiration (FNA) of the mass. According to a large retrospective study published in 2023, FNA’s accuracy is 96.5%, and its sensitivity, specificity, positive predictive value and negative predictive value are 94.4%, 98.2%, 97.7% and 95.6%, respectively [2].

The mainstay of treatment for PA is surgical resection of the tumor. Since most PAs originate in the superficial lobe of the parotid gland, superficial parotidectomy usually suffice for complete resection. Care must be taken to not violate the tumor’s capsule, to decrease the risk for recurrence. Recurrence rate is generally low. In an analysis performed on 5497 PA patients from the Danish pathology bank data in 2015, the recurrence rate was 2.86% [3]. Other, higher rates were reported as well [4].

The main anatomic structure to watch for and preserve during surgery is the facial nerve. Although there are no *real* superficial and deep parotid lobes, and thus, it is customary to divide the gland according to the plane the facial nerve lies in. During parotidectomies, the facial nerve is usually exposed at its main trunk, emerging out from the skull base to the parotid tissue. The common landmarks used when exposing the facial nerve are the tympanomastoid suture line (the most consistent landmark); the plane of the digastric muscle—the nerve emerges between the muscle and the styloid process; and to look for the tragal pointer—the nerve is normally found 1–2 cm deep and inferior to the tragal pointer. Other less commonly used methods in benign tumors are performing a mastoidectomy to expose the nerve’s entire course and to perform a retrograde dissection—locating one of the nerve’s peripheral branches, such as the marginal mandibular or cervical nerve, and dissecting back to the nerve’s main trunk [1].

Treatment of recurrences depends on many factors including but not limited to patient characteristics such as comorbidities or various anesthesia considerations; recurrence characteristics, for example, single vs. multiple tumors identified; primary surgery characteristics: Did a violation of the tumor’s capsule occur? Or was there tumor spillage?

Treatment options include revision surgery, radiotherapy and follow-up in specific selected patients, most of whom have many comorbidities, making surgery and radiotherapy less favorable options.

Radiotherapy is usually offered to patients who have had seeding of the tumor in their parotid bed, resulting in multiple small PA lesions; to patients with high risk for facial nerve damage during revision surgery; patients unfit for surgery.

In a study from 2006 by Chen et al., a local control rate of 94% for patients treated with radiotherapy for recurrent PA was demonstrated [5]. Another important study by Nicholas et al. demonstrated that adjuvant radiotherapy was associated with significantly decreased risk of recurrence with a hazard ratio of 0.09% compared to surgery alone [6].

Due to its benign nature, there are almost no guidelines published for routine follow-up of PA after surgery, but due to the alleged malignant transformation risk, routine follow-up is customary. It should be noted that the French society of Otorhinolaryngology–Head and Neck surgery published guidelines regarding recurrent PA. They state that patients suspected with recurrence should undergo an MRI and that more extensive surgery should be performed for recurrent disease [7].

In today’s medical environment, cost-saving evidence-based medicine is crucial for health organizations. Possible futile long-term follow-up plans, especially for benign pathologies, need to be addressed and considered while balancing possible costs with potential benefits.

The aim of this study was to describe our experience regarding PA recurrence, to examine the need and yield for routine follow-up after PA removal, its frequency and duration and the cost–benefit value of such follow-up.

## 2. Materials and Methods

### 2.1. Study Design

A retrospective analysis of all patients who had undergone resection of PA confirmed by pathology at a university-affiliated tertiary care center between 1990 and 2023 was performed.

Research ethics: the study was approved by the Institutional Ethics Review Board, granting a waiver of informed consent. Authorization number RMC-24-0794.

### 2.2. Variables and Measurements

Data regarding patient’s surgery details such as date, type of surgery, subsite, pathology, means of follow-up (office, imaging, etc.), number of office examinations per patient/tumor presentation, number and type of imaging studies performed for each patient, data regarding tumor recurrence including date, time from treatment to first recurrence, method of recurrence detection and number of recurrent events per patient were collected and analyzed. Patients with insufficient medical records data regarding recurrence detection method were proactively called and questioned.

Data was collected primarily from our institution electronic medical records (EMRs). More data was acquired through patients’ HMO records, when available. While access to office examinations outside the hospital is not always available, most imaging studies’ results are available in the HMO records that we have access to.

Prices in USD (United States Dollar, converted from New Israeli Shekel) for doctor’s office appointment, MRI and US were collected according to Israel’s Ministry of Health price list [8].

Prices include the following: Doctor’s office appointment—88 USD; MRI—635 USD; Neck US—121 USD.

All US were performed by trained and certified imaging specialists; all MRIs were interpreted by experienced neuroradiologists.

### 2.3. Our Center’s Protocol for PA Diagnosis and Treatment

Every patient who presents with a mass in their parotid gland is examined and evaluated as follows:

- Full ENT history and examination by a fellowship trained head and neck surgeon, with emphasis on mass characteristics and facial nerve function.
- Ultrasonography.
- FNA.
- MRI as needed. Usually we use MRI for the following purposes: large mass; rapidly enlarging mass; suspicion for malignancy in patient’s history or physical examination; suspicion of involvement of sites other than the superficial lobe of the parotid gland.
- Other inquiries as needed per patient.

Patients usually undergo surgery within 3 months of their first examination by a specialist. All surgeries are performed by a fellowship trained head and neck surgeon with extensive experience in parotidectomies. The minimal surgery patients undergo is superficial parotidectomy, extended as needed per case.

We do not perform enucleation due to extremely high recurrence rates.

During surgery, the facial nerve is exposed and guarded meticulously.

### 2.4. Our Center’s Protocol for PA Follow-Up

As mentioned before, follow-up is managed according to the surgeon’s discretion, but most surgeons manage PA patients’ follow-up according to the following protocol:

- At 4–6 weeks after surgery, follow-up for surgical site examination and pathology report.
- Once yearly for 5 years after surgery, with up-to-date US.
- Patients are encouraged to report any new symptoms between follow-up meetings.
- MRI is not used routinely. It is used based on surgeon’s discretion, mainly after suspicious US results during follow-up.

### 2.5. Statistical Analysis

Data analysis was performed with IBM SPSS statistics for Windows, version 27 (IBM Corp., Armonk, NY, USA).

Descriptive statistics: Categorical variables were expressed as frequency and percentages. Continuous variables were expressed as mean and standard deviation (SD) or with range (minimum–maximum) if normally distributed or as median with interquartile range (Q1, Q3) if non-normally distributed.

## 3. Results

### 3.1. PA Recurrence Rate and Time to Recurrence

**Figure 1:**
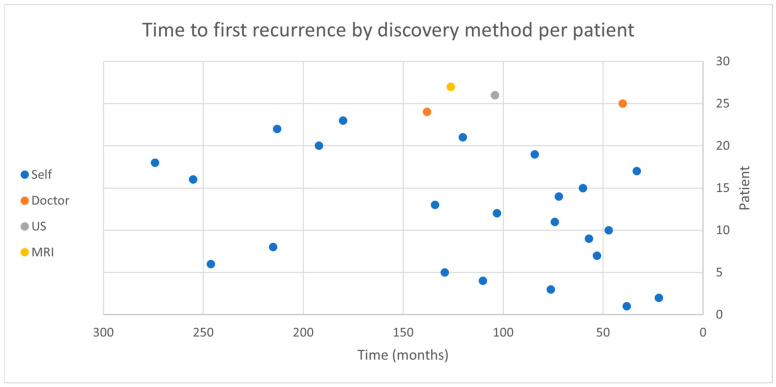
Depicts the detection method and time of each pleomorphic adenoma recurrence per patient. Each dot represents the first recurrence detection method.

Overall, 301 patients had undergone surgery for PA removal during this period. A total of 28 of them experienced recurrence (9.3%), 9 of them with multiple events (range 2–8). Mean time to first recurrence was 119.5 months (range, 33–274). First recurrence method of detection and time to recurrence is depicted in Figure 1. Patients’ demographics are depicted in Table 1.

### 3.2. Recurrence Detection Method and Time to Recurrence

Of the 40 recurrences, 36 were self-discovered (90%), 2 were discovered during routine follow-up visit (5%) and 2 by imaging (5%). Of the two discovered by imaging, one was discovered by MRI, and the other one was discovered by pre-planned regular follow-up US.

The earliest recurrence was detected 33 months after surgery (1/28, 3.5%) by self-palpation. All other first recurrences occurred after 40 months or more.

Overall, three patients (3/28, 10.7%) had an unvalidated mode of first recurrence detection, and three more with multiple recurrences (3/9, 33%) had insufficient data regarding the method of re-recurrence detection.

Therefore, phone calls were made to three patients with multiple recurrences—all of them discovered each disease recurrence themselves by palpation and scheduled an appointment to our clinics.

Three more patients with validated recurrence but insufficient records were contacted by phone regarding their recurrence. All patients who were contacted by phone had their recurrence at least 8 years ago. All six patients who were reached by phone answered clearly and without hesitation that they discovered the recurrence by self-palpation.

The missing data we asked for was regarding the method of recurrence detection—a simple question was asked, as follows: “How did you discover the recurrence of disease?”

### 3.3. Follow-Up Office Examinations and Imaging Studies Cost Description

A total of 618 office examinations were performed in the entire group, 152 (24.5%) of them were patients who had recurrence. The six hundred and eighteen office examinations cost approximately 55,000 USD.

Three hundred and nine office examinations were conducted for one recurrence detection. One hundred and fifty-five imaging studies were conducted overall: 54 (34%) in the recurrence group, 41 of them MRIs and 114 USs. Seventy-seven imaging studies were performed for one recurrence detection.

The 41 MRI studies cost approximately 26,500 USD, and the 114 US studies cost approximately 14,000 USD.

### 3.4. Facial Nerve

In our cohort, only 17 patients (5.6%) had facial nerve weakness which appeared immediately after surgery, assessed by physical examination. None of them had complete paralysis, and over 80% of them had a mild weakness with a House Brackmann score of 2–3. On long-term follow-up, facial nerve function recovered completely in 15 patients (88%), one remained with mild facial nerve weakness, and the last one was lost to follow-up and his facial nerve status is unknown.

### 3.5. Parotidectomy Type

Two hundred and fifty-one patients underwent superficial parotidectomy. Thirty-six underwent subtotal parotidectomy. Recurrence rates were similar between the groups (8.3% vs. 9.2%); Fisher’s exact test *p* = 1.00.

## 4. Discussion

In this study, we investigated the need for routine follow-up after PA resection and the cost–benefit value of such follow-up. We have shown, in a large cohort, that the need for routine follow-up is questionable, and that substantial amount of money can be saved if patient-oriented follow-up approach is implemented.

Existing data in the medical literature remains scarce regarding the method of PA recurrence detection. In a study from 2008, Redaelli de Zinis et al. described that 29/33 recurrences were discovered by the patient [9]. Another study by Ayoub et al. found only one self-discovered recurrence in 58 patients followed for a mean period of 6 years. The authors concluded that patient education can replace long-term follow-up [10]. It is also noteworthy to mention that the presenting symptom in most primary cases of PA is parotid swelling, rather than pain or incidental imaging finding [11].

Our study showed that patients detect recurrences themselves in most cases. Even patients with multiple recurrences who logically “deserve” follow-up by imaging, discover their recurrences mainly by themselves. This fact was ratified by phone calls to patients whose medical records were indeterminate regarding method of recurrence detection. Although these calls are prone to recall bias, we asked a specific question regarding the method of recurrence detection—all patients recalled this information easily and clearly.

Several studies in the past have shown that proper resection techniques of PA result in very few recurrences. Several factors contribute to recurrences: violation of the tumor’s capsule and spillage, failure to resect healthy parotid tissue around the tumor and other factors such as size and myxoid subtype [12].

Although superficial parotidectomy is the common mainstay of treatment, several surgical techniques have been described for PA resection.

In the past, enucleation of the tumor was customary. Enucleation consists of dissection on or within the tumor capsule, with no resection of marginal normal parotid tissue. Studies from the 20th century have described an unacceptable rate of recurrence associated with enucleation. A study conducted in Germany in the 1990s reported a recurrence rate of 14% [13].

In a systematic review published in 2017, rates as high as 40% (!) were described [12]. Therefore, enucleation was discarded as a surgical option for pleomorphic adenoma.

The reason for this high rate of recurrence with enucleation is probably the tumor’s pseudopodia present in 25% of PAs and its thin capsule which is missing focally around the tumor in 50% of PAs [14]. When a surgeon attempts enucleation of the tumor, any one of these histopathologic features can hinder the tumor’s complete resection and raise the probability of recurrence. In our study, recurrence rate was 9.3%.

We believe this recurrence rate is because of two main factors: (1) Our cohort consists of patients operated in the 1990s—surgical technique, facial nerve monitoring and the ability to handle complex cases have evolved greatly since then. (2) As elaborated hereinafter, the time to recurrence in PA cases is long. For this reason, studies such as ours spanning over 30 years of follow-up will surely show more recurrences than studies with significantly shorter follow-up times.

Moreover, the time to recurrence in PA is long. Many studies have addressed this issue. For example, Lombardi et al. found that the median time to recurrence was 54 months [15]. In a randomized controlled trial from 2007, no recurrences were found during a 4-year follow-up period [16]. Based on two nationwide series of PA, in a mean follow-up of 12.5 years, only 3.1% of patients developed recurrence [17]. Our study showed similar results with a mean time to first recurrence of 119.5 months. Thus, a follow-up plan consisting of many years of office examinations and multiple US studies seems low-yield. In our cohort, over 90% of patients discovered the first recurrence by themselves as depicted in Table 1. These results underscore the importance of patient education and also show that for most patients, imaging studies are not required for routine follow-up.

In our institution, follow-up is managed according to the surgeon’s discretion, but most surgeons conduct follow-ups with patients annually, along with routine office appointment and ultrasonography (US) exams.

Limited data exist regarding the true need for long-term follow-up after surgery and the detection method of recurrence. For instance, in an article from the UK, the only recurrence recorded, was self-discovered [10]. Other studies proposed a follow-up plan, but it is not evidence-based [15]. The mean time to recurrence was examined in many studies, some with results of up to 10 years until recurrence [18]. Nevertheless, it seems that the scarce follow-up plans found in the literature fail to take PA’s recurrence timeline into account.

An argument advocating routine follow-up could be made based on the malignant transformation potential of PAs. While our study’s aim was not to collect data regarding malignant transformation potential, other notable recent studies have performed as such.

The risk for malignant transformation for a patient diagnosed with PA was traditionally described as 1.5% within the first 5 years of diagnosis and 10% in 15 years [1]. A recent study has challenged these numbers, stating malignant transformation rate at 3.2% in untreated PA cases with a median follow-up period of 3.2 years, with no difference in incidence between groups of varying follow-up length. It also repeated a notion raised in previous studies: it is uncertain whether malignant *transformation* happens over time or that malignancy is present *from the outset* [19].

In a study published by Levyn et al. in 2023 regarding the duration of follow-up of untreated PA, the duration of follow-up was not associated with malignancy [19]. Another study published in 2001 found that nearly half of the patients diagnosed with ex-pleomorphic adenoma malignant tumors had signs/symptoms for less than a year before diagnosis [20].

As the literature is abundant with studies describing risk factors for recurrence after excision of PA, we did not attempt to study this issue but rather focused on the timeframe and method of recurrence detection. The main risk factors identified in several studies are young age at presentation, tumor spillage during surgery and tumor size [5,12,13].

Another issue to which no reference was found is the economic aspect of long-term follow-up. We found that most office appointments and imaging studies are futile for routine follow-up. Since PA is the most common salivary gland tumor, large tertiary centers operate on many patients per year. Due to the nature of the disease, patients might have dozens of follow-up office visits over the years. To say the least, hundreds of thousands of dollars can be saved each year by tailoring an individualized follow-up plan for each patient. This is also true for the patients themselves. Although in our country most imaging studies are funded by the HMO, some may require co-payment by the patient. In addition, loss of work hours and travel expenses are also an important consideration for patients and their well-being.

For example, patients who have had tumor spillage during surgery should be followed-up more frequently than those who did not. However, patients who had an uneventful surgery, and in whom pathology has shown complete tumor resection with negative margins, may not be followed-up after the first post-operative office visit. Moreover, during the first 3 years following surgery, it seems that neither imaging nor office examination is necessary for a “standard” patient, and most patients can be educated post-operatively to seek medical attention if any lump or other bothersome signs/symptoms become apparent. Patients should also be educated about Frey syndrome (gustatory sweating) and its management, because many patients may seek medical attention for these symptoms. However, it should be stated that in any case of suspected recurrence, an MRI should be performed to assess the extent of disease and to guide the surgical approach for revision surgery, as stated in the French guidelines for recurrent PA [7]. With all of the above in mind, the importance of meticulous, sound surgical technique and experienced fellowship trained surgeons performing the surgery cannot be overstated. With that in mind, when patients who were operated by less experienced surgeons seek medical attention, increased vigilance is required by the otolaryngologist. Performing a “baseline” US and perhaps even an MRI, depending on the circumstances and characteristics of the patient and the surgery he underwent is plausible. Revision surgeries in such cases can be even more challenging then “normal” revision surgeries as the surgical technique used might lack the high standards employed by high volume centers.

Several studies were published in the past with large differences in the rate of facial nerve weakness after parotidectomy for benign pathologies. One study reported a remarkable 46% weakness rate. Nevertheless, it is widely accepted that permanent facial nerve weakness after parotidectomy is rare and that most patients will recover completely. Revision surgeries, operating in a radiated field and parotidectomies involving the deep lobe of the parotid are more prone to facial nerve injury [1,21,22,23,24].

Even though a study from the UK published in 2007 which directly compared parotidectomies for between ENT and non-ENT surgeons found no difference with regard to complications and specifically facial nerve palsy, it is clear that the non-ENT surgeons who conducted the surgeries were trained professionals [25]. It makes complete sense that as long as the surgeon is well familiar with the anatomy and surgical steps and has enough surgical experience, he will reach similar results as the ENT surgeon.

One main limitation of our study is its retrospective nature and that it is based on the records of our institution, spanning three decades. Unfortunately, the medical records are not comprehensive enough and we assume that many follow-ups and imaging studies remain unknown. Furthermore, many patients decided to continue their follow-up in the private sector, or with their health maintenance organization (HMO), thus resulting in incomplete data. This may have led to underestimation of the number of office examinations and imaging studies performed for follow-up and their financial impact, thus causing the effect on detection yield to be uncertain.

Another important limitation is our inability to refer to patients’ educational level and socioeconomic status due to the retrospective nature of the study. It is imperative for the surgeon to assess all the intricate aspects of the patient and not only his disease. Uneducated patients from a lower socio-economic background may not adhere to the surgeon’s recommendations and might show up only after a recurrence has become a major problem esthetically or functionally. Thus, patients from such backgrounds should be followed-up more regularly than others.

Finally, the fact that the time period is so long means that patients had their surgeries performed by several head and neck surgeons. Although they are all qualified and certified surgeons, surgical techniques and equipment have changed and evolved over the years.

Other confounding factors are the quality of US equipment and MRIs’ sensitivity which have improved substantially in the study’s time period, which may have resulted in a potential bias resulting in underdiagnosis many years ago.

## 5. Conclusions

To conclude, a patient-oriented follow-up plan considering the patient’s age, tumor and surgery characteristics is advised. Patients whose surgeries involved tumor spillage, tumor’s capsule was violated, or large tumors which extended further from the parotid to the parapharyngeal space, for instance, should be considered high-risk patients and followed-up accordingly. Patients’ adherence and probability of seeking medical care should a recurrence be suspected must also be taken into consideration. Almost all recurrences in our study and those reported in the literature were discovered more than 3 years after surgery, thus routine follow-up with office examinations or imaging prior to this timeframe seems to be of low yield in most instances, and as outlined above, malignant transformation, if plausible, is exceedingly rare. Patients should be counseled about ominous symptoms necessitating medical care such as new swelling, facial nerve palsy or pain in the surgical bed. In such circumstances, an US and an MRI should be obtained promptly. We strongly believe that such a strategy is both safe and cost-effective.

## Figures and Tables

**Table 1:** Demographic and clinical parameters. US—Ultrasonography.

Parameter	Study Group (*n* = 301)
**Age at first surgery (years)**	49.87 (±17.28)
**Gender**	
**Male**	114 (38%)
**Size on US pre-op (cm)**	2.11 (±0.9)
**Total follow-up office examinations**	2.87 (±2.7)
**Total number of US**	1.36 (±1.49)
**Smoking**	65 (21.5%)
**Side**	
**Right**	155 (54.4%)
**At least one recurrence**	28 (9.3%)
**Parotidectomy type and recurrence rate**	
Superficial	251 (83.3%)
**Recurrence rate**	23 (9.2%)
Subtotal	36 (11.9%)
**Recurrence rate**	3 (8.3%)
Data unavailable	14 (4.6%)
**Recurrence rate**	1 (7.1%)
**Multiple recurrences**	9/28 (32%)
**Recurrence detection method—Total (*n* = 40)**	
Self	36 (90%)
Imaging	2 (5%)
Office exam	2 (5%)
**Recurrence detection method—First (*n* = 28)**	
Self	26 (93%)
Imaging	1 (3.5%)
Office exam	1 (3.5%)
**Time to first recurrence (months)**	119.5 (±71.9)
**Time to first recurrence range (months)**	33–274
**Facial Nerve injury**	17 (5.6%)
House Brackmann score 2–3	14 (82.3%)
House Brackmann score 4–5	3 (17.6%)

## Data Availability

The data that support the findings of this study are available upon reasonable request from the corresponding author. The data are not publicly available due to privacy or ethical restrictions.
